# Comprehensive Phytohormone Profiling of Kohlrabi during In Vitro Growth and Regeneration: The Interplay with Cytokinin and Sucrose

**DOI:** 10.3390/life12101585

**Published:** 2022-10-12

**Authors:** Tatjana Ćosić, Václav Motyka, Martin Raspor, Sumbal Sajid, Nina Devrnja, Petre I. Dobrev, Slavica Ninković

**Affiliations:** 1 Department of Plant Physiology, Institute for Biological Research “Siniša Stanković”—National Institute of Republic of Serbia, University of Belgrade, Bulevar Despota Stefana 142, 11060 Belgrade, Serbia; 2 Laboratory of Hormonal Regulations in Plants, Institute of Experimental Botany of the Czech Academy of Sciences, Rozvojová 263, 16502 Prague 6, Czech Republic; 3 School of Life Science and Engineering, Southwest University of Science and Technology, Mianyang 621010, China

**Keywords:** kohlrabi, cytokinin, sucrose, in vitro development, shoot regeneration, phytohormone profiling

## Abstract

The establishment of an efficient protocol for in vitro growth and regeneration of kohlrabi (*Brassica oleracea* var. *gongylodes*) allowed us to closely examine the phytohormone profiles of kohlrabi seedlings at four growth stages (T1–T4), additionally including the effects of cytokinins (CKs)—*trans*-zeatin (*trans*Z) and thidiazuron (TDZ)—and high sucrose concentrations (6% and 9%). Resulting phytohormone profiles showed complex time-course patterns. At the T2 stage of control kohlrabi plantlets (with two emerged true leaves), levels of endogenous CK free bases and gibberellin GA_20_ increased, while increases in jasmonic acid (JA), JA-isoleucine (JA-Ile), indole-3-acetic acid (IAA) and indole-3-acetamide (IAM) peaked later, at T3. At the same time, the content of most of the analyzed IAA metabolites decreased. Supplementing growth media with CK induced de novo formation of shoots, while both CK and sucrose treatments caused important changes in most of the phytohormone groups at each developmental stage, compared to control. Principal component analysis (PCA) showed that sucrose treatment, especially at 9%, had a stronger effect on the content of endogenous hormones than CK treatments. Correlation analysis showed that the dynamic balance between the levels of certain bioactive phytohormone forms and some of their metabolites could be lost or reversed at particular growth stages and under certain CK or sucrose treatments, with correlation values changing between strongly positive and strongly negative. Our results indicate that the kohlrabi phytohormonome is a highly dynamic system that changes greatly along the developmental time scale and also during de novo shoot formation, depending on exogenous factors such as the presence of growth regulators and different sucrose concentrations in the growth media, and that it interacts intensively with these factors to facilitate certain responses.

## 1. Introduction

As sessile organisms, plants must perceive and understand various signals, both internal and external from their environment, and carefully transmit them to specific intrinsic machinery. This machinery utilizes numerous and diverse control and regulatory pathways that interact with each other to facilitate the proper responses and thus all of the morphological and physiological processes underlying plant development. Plant hormones play an essential role in this complicated system [1]. They are characterized by distinct biosynthetic and catabolic pathways, and their metabolism and action are regulated both temporally and spatially and may influence each other’s synthesis and activity. Phytohormones often undergo specific modifications, such as conjugation with various metabolites, e.g., amino acids or glucose, methylation, or hydroxylation, all of which affect their biological activity in terms of enhancement or reduction [2,3,4]. Thus, in addition to the predominant active forms of phytohormones, there are also many different related compounds, such as inactive precursors, additional active forms, storage and conjugated forms, etc., that act together in regulating the levels of active hormones in plants. These particular compounds also function as signaling molecules, integrating and balancing developmental processes through a sophisticated and multifaceted setup [5,6].

Starting from the “basic” or essential auxin and cytokinin (CK) groups, quite different collections of phytohormones and their related metabolites have emerged according to current knowledge, all of which have specific functions for plant growth and behavior. Auxins, as well as CKs and gibberellins (GAs), are primarily associated with plant developmental processes and, based on their earlier discovery and characterization, are often referred to as growth phytohormones [7] or sometimes even as “classical” phytohormones, along with abscisic acid (ABA) and ethylene [8]. On the other hand, the latter two groups, along with jasmonic acid (JA) and salicylic acid (SA), have been referred to as stress/defense hormones for some time. However, numerous studies have demonstrated that these so-called stress hormones can be involved in various developmental aspects of plant life and vice versa, obscuring the line between these two groups and recognizing their miscellaneous and multiple roles in plant organisms (reviewed in [9]).

Given the ability of plants to translocate auxins not only via long-distance phloem transport but also to utilize complex directional cell-to-cell polar transport, auxins are thought to play an essential role in the final shaping of plant cells and tissues and to significantly influence their function [10,11,12]. Many of the auxin actions are interconnected with CKs, a group of plant regulators that control plant cell cycle, embryo development, photomorphogenesis, and leaf senescence, among others, including their role in enhancing plant responses to environmental stress [13,14]. Since the discovery that the proper ratio of auxin and CK can determine the developmental fate of cells, implying that callus differentiation is induced to form either roots or shoots [15], appropriate in vitro manipulations of these plant hormones have been widely used to regenerate whole plants from undifferentiated callus tissue, providing the basis for the development of plant biotechnology.

As with auxins and CKs, homeostasis of GA is closely feedback-regulated by its own metabolism and signaling [16]. Of 136 different gibberellin-like compounds discovered, only a few, such as GA_1_, GA_3_, GA_4_, and GA_7_, are considered bioactive [17] and are responsible for promoting cell division and elongation, seed germination, flowering induction, pollination, and plant adaptation to stresses [18,19,20].

Previously considered primarily a growth inhibitor and stress phytohormone, ABA is now recognized as a key compound for numerous physiological processes [21]. Similarly, JA and SA not only play important roles in regulating plant stress responses [22,23], but are also involved in seed germination, root formation, leaf senescence, and flowering [24,25]. JA and SA pathways are often intertwined and usually antagonistic in many plant species [22]. Multiple interactions with other phytohormones to appropriately regulate various processes such as ripening stimulation and senescence, as well as responses to biotic and abiotic stresses, may also be associated with the gaseous phytohormone ethylene [26]. Recently, it was shown that 1-aminocyclopropane-1-carboxylic acid (ACC), the direct precursor of ethylene, may also have a distinct signaling function in regulating plant development (reviewed in [27]).

It is well known that phytohormone and sugar signaling are closely linked in higher plants, controlling various developmental processes and influencing each other’s levels, transport mechanisms, and localization [28,29,30]. There are numerous mechanisms in plants responsible for fine-tuning processes controlled by hormones to match the carbon status of the plant, of which sugar sensitivity is an essential part [31]. Sugars are not only a source of energy, but also serve as signaling molecules that influence various elements of phytohormone-activated metabolic pathways that regulate different aspects of plant life [32,33]. These interactions occur at transcriptional, posttranscriptional, and posttranslational levels [29,34]. The crucial role in regulating plant growth relates to the proper coordination of signaling pathways that respond to metabolic, hormonal, and environmental stimuli. For example, hexokinase 1 (HXK1) has been shown to influence glucose, auxin, and CK-related signaling pathways [32], making the regulatory network even more complex.

Kohlrabi (*Brassica oleracea* var. *gongylodes*) is a vegetable crop that belongs to the *Brassica* genus, well known for its edible tuberous stems that are rich in vitamins, minerals, and fibre. Kohlrabi in vitro culture has been established and effectively used in our laboratory as a model system for the study of in vitro plant morphogenesis [35,36,37,38,39]. A one-step shoot regeneration protocol has been developed for de novo shoot organogenesis (DNSO) of kohlrabi using intact seedlings cultured on different SIM (shoot induction medium) formulae consisting of the growth media only supplemented with CKs as explants [36]. Histological and expression analyses showed that organogenesis in kohlrabi followed the same sequence of morphogenic events as in the standard two-step regeneration of *Arabidopsis* even without preincubation on auxin-rich CIM (callus induction medium) [36,37]. Application of different sugars and plant growth regulators (PGRs) also showed their differential effects on in vitro growth and development of kohlrabi seedlings and altered various morphological traits compared to corresponding controls [38]. Recently, our research group also indicated a possible crosstalk of sucrose and phytohormones during DNSO in kohlrabi seedlings and showed significant impact of both CK and sugar treatments and their interplay, suggesting that sucrose signaling may interact with phytohormone homeostasis [39].

The aim of this study was to further investigate kohlrabi growth and development in vitro, focusing on kohlrabi phytohormonomics. Using HPLC–ESI–MS/MS and principal component analysis (PCA), we have, for the first time, determined the phytohormone profile of four growth stages (T1–T4) of in vitro cultured kohlrabi, cv. Vienna Purple, with additional consideration of the effect of CKs (*trans*-zeatin, *trans*Z; thidiazuron, TDZ) and high sucrose administration (6% and 9%).

## 2. Materials and Methods

### 2.1. Plant Material

#### 2.1.1. Seed Sterilization

Kohlrabi (*Brassica oleracea* var. *gongylode*s cv. Vienna Purple) seeds were first immersed in 70% ethanol for 5 min and then in 30% commercial bleach (4–6% NaOCl) containing a drop of detergent (Fairy; Procter and Gamble, London, UK) for 30 min, according to the method described previously [39]. They were then thoroughly rinsed in autoclaved distilled water. After sterilization, seeds were aseptically transferred to specific growth media.

#### 2.1.2. Growth Media and Conditions

Basal, hormone-free growth medium (MS) consisted of MS mineral salts [40], vitamins [41], 3% sucrose, 100 mg·L^−1^ inositol, solidified with 0.6% agar. In order to study the effects of sucrose and exogenous CKs, MS media containing 6% or 9% sucrose and MS media supplemented with *trans*-zeatin (*trans*Z) or thidiazuron (TDZ) at 2 mg·L^−1^ were used, respectively. Additionally, media containing a combination of distinct CKand 6% or 9% sucrose were used. The applied sucrose and CK concentrations were previously determined by optimizing the regeneration protocol, as described earlier [36,39]. The pH of all media used in this study was adjusted to 5.8 using 1 N NaOH prior to autoclaving at 114 °C and 80 kPa for 25 min. The in vitro cultured plants were maintained at a temperature of 25 ± 2 °C under cool white fluorescent light with an irradiance of 47 µmol m^−2^·s^−1^ (16/8 h light/dark photoperiod).

#### 2.1.3. Experimental Setup

The experiment was performed at the Department of Plant Physiology at the Institute IBISS in Belgrade, Serbia. To evaluate the endogenous hormone content of kohlrabi and the influence of sucrose and CKs on endogenous hormone homeostasis during four developmental stages (T1–T4) of kohlrabi seedlings, eight laboratory jars (375 mL), each containing 6 sterilized seeds, were used for separate treatments, including the control grown on MS. The developmental stages studied included: T1-seedlings bearing two cotyledons; T2-plantlets with emerged two true leaves; T3-plantlets forming callus at the base of the stem; and T4-calli bearing de novo shoots. As determined earlier [36,38,39], during treatment with CK as well as elevated sucrose concentration, kohlrabi seedlings tend to form callus at the base of the stem, eventually leading to de novo forming shoots under the influence of the CKs. Control seedlings/plantlets grown on basal growth medium, that genuinely lack any form of regeneration, were sampled at the same time as the equivalent CK treated plantlets. For each developmental stage, three representative plantlets exposed to the same treatment were collected and pooled into a single biological sample. Collected samples were instantly frozen in liquid nitrogen and stored at −70 °C until analysis. The complete experimental setup was performed in three independent biological replicates.

### 2.2. Phytohormone Extraction, Purification and Quantification

The phytohormone analysis was performed as previously described [42,43]. Primarily, about 100 mg of collected plant material from each biological sample was frozen and homogenized in liquid nitrogen. After lyophilisation, 500 μL of cold extraction buffer (methanol/formic acid/water, 15/1/4; *v*/*v*/*v*) and a mixture of stable isotope-labelled internal standards (10 pmol) were added to the plant homogenates. The following internal standards were used for the analysis: [^13^C_6_]IAA (Cambridge Isotope Laboratories, Tewksbury, MA, USA); [^2^H_4_]salicylic acid (SA) (Sigma-Aldrich); [^2^H_3_]phaseic acid (PA; NRC-PBI, Saskatoon, SK, Canada); [^2^H_5_]jasmonic acid (JA; C-D-N Isotopes Inc., Pointe-Claire, QC, Canada); [^2^H_6_]ABA (NRCPBI); [^2^H_5_]*trans*-zeatin (*trans*Z); [^2^H_3_]dihydrozeatin (DHZ); [^2^H_6_]*N^6^*-(∆^2^-isopentenyl)adenine (iP) (OlChemIm, Olomouc, Czech Republic). The content of *cis*-zeatin (*cis*Z) was determined based on the retention time and mass spectrum of the unlabeled standard and the response ratio of its *trans*Z counterpart.

Phytohormone detection and quantification were carried out using HPLC (Ultimate 3000, Dionex, Sunnyvale, CA, USA), coupled to a hybrid triple quadrupole/linear ion trap mass spectrometer (3200 Q TRAP, Applied Biosystems, Foster City, CA, USA) set in selected reaction-monitoring mode. Reversed phase and ion-exchange chromatography (Oasis-MCX, Waters, Milford, MA, USA) was exploited for acquiring fraction A, eluted using methanol, and fraction B, eluted using 0.35 M NH_4_OH in 70% methanol.

The negative mode of the mass spectrometer electrospray ionization was set for fraction A, which contained auxins, ABA, SA, JA and their derivatives (acidic and neutral hormones), while the positive mode was set for fraction B, which contained CKs and 1-aminocyclopropane-1-carboxylic acid (ACC) (alkaline hormones). The obtained fractions were evaporated to dryness in a vacuum concentrator and solvated in 30 mL of 10% methanol. The mass spectrometer was set at electrospray ionization mode with ion source voltage −4000 V (negative mode) or +4500 V (positive mode); nebuliser gas 50 psi; heater gas 60 psi; curtain gas 20 psi; heater gas temperature 500 °C. Quantification of the phytohormones was performed via isotope dilution method with multilevel calibration curves. All data were processed using Analyst 1.5 software (Applied Biosystems) and the concentrations of phytohormones were calculated as quantity per 1 g of fresh weight plant material. Full names and abbreviations along with molecular structure of analyzed phytohormones are presented in Supplementary Figure S1.

### 2.3. Data Analyses

Data were analyzed using SAS software (SAS Institute, 2002. SAS/STAT, ver. 9.00. SAS Institute Inc., Cary, NC, USA). Results were presented as mean values of three biological replicates ± standard error (SE). Student’s *t*-test was performed to demonstrate the difference of individual treatment compared to control for each developmental stage examined. The statistical correlation between the levels of the main phytohormone group representatives and the levels of their distinctive metabolites was evaluated for each developmental stage and presented using Pearson’s correlation coefficient (r). Principal component analysis (PCA) was conducted in the FactoMineR R package (R version 4.0.3) [44]. For the purpose of PCA, the data measured in the present work were divided into four groups, which were presented as data measured for individual developmental stages (T1–T4), in order to determine variability of the kohlrabi phytohormonome between different culture treatments. Additionally, the data were divided into 5 groups corresponding to each treatment applied to determine variability over the time-course of seedling development. In addition, the ratio between the content on a medium containing a particular CK with 6 or 9% sucrose and a CK medium with 3% sucrose was determined for all metabolites analyzed and presented as a joint heat map.

## 3. Results

We investigated in detail the phytohormone content of four stages (T1–T4) during in vitro growth and development of kohlrabi seedlings. In addition, this study included the effect of exogenously added CKs (*trans*Z and TDZ) or high sucrose concentrations (6% and 9%). Supplementing growth media with CK or sucrose induced changes in kohlrabi development, particularly in later stages, marked as T3 and T4. The plantlets were shorter with thicker stem, especially when 9% sucrose or TDZ was applied. While callus formation at the base of the stem occurred during both type of treatments, only CKs led to development of de novo shoots [39]. Depending on the phytohormone types and/or their metabolites studied, individual changes could be observed on a growth time scale resulting in particular hormone profiles.

### 3.1. Auxins

**Figure 1:**
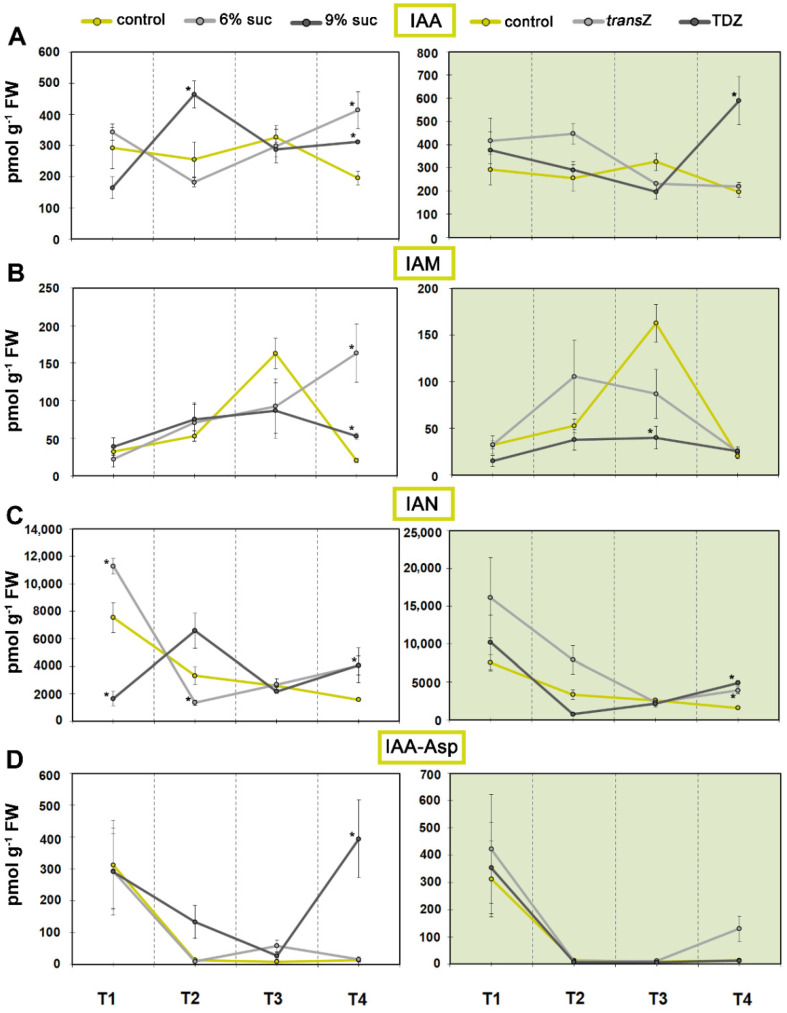
Changes in endogenous levels (in pmol g^−1^ FW) of indole-3-acetic acid (IAA) and related metabolites at four developmental stages (T1–T4) during in vitro growth of kohlrabi (cv. Vienna Purple) on media supplemented with 3(control)/6/9% sucrose or 2 mg L^−1^ *trans*Z/TDZ. (**A**) IAA; (**B**) indole-3-acetamide (IAM); (**C**) indole-3-acetonitrile (IAN); (**D**) IAA-aspartate (IAA-Asp). Results are expressed as mean ± SE (*n* = 3 independent biological replicates). Means marked with * are significantly different from control according to Student’s *t*-test (*p* < 0.05) at each developmental stage. *trans*Z: *trans*-zeatin; TDZ: thidiazuron.

Little variation in indole-3-acetic acid (IAA) levels were observed throughout the development of the kohlrabi control plants, except at the T3 stage, where the IAA content was transiently elevated. After exposure to 9% sucrose a significant increase in concentration was observed at T2, whereas both sugar treatments induced IAA accumulation at the T4 stage (Figure 1A). Of the exogenous CKs, only TDZ induced an increase in IAA content at the final growth stage, T4. In contrast to IAA itself, IAA metabolites generally showed differentially patterned changes during the studied kohlrabi development of the control plants. Indole-3-acetamide (IAM), the precursor of IAA, showed a strong increase at T3 (~5-fold), and reached maximum before decreasing to the initial level at T4. However, cultivation on elevated sucrose resulted in IAM content being significantly higher than the control value at T4. TDZ, on the other hand, had the opposite effect and lowered the IAM levels, particularly at T3 (Figure 1B). For indole-3-acetonitrile (IAN), another IAA precursor, a continuous decrease was observed in the control as development progressed, lowering the level at T4 by 4.8-fold compared to the initial value. At the first two growth stages, different sucrose concentrations showed an opposite effect on the levels of IAN, which subsequently consolidated. Both CKs showed statistically significant effects only at T4 (Figure 1C). The profiles of IAA-aspartate (IAA-Asp), one of the most abundant auxin conjugates, were generally quite similar regardless of the treatment applied, with maximum values reached at the beginning of the developmental time scale, followed by a sharp decrease of more than 20-fold at T2 and at the other stages analyzed. An exception was T4, after the highest sucrose concentration had been used, when the content exceeded the value recorded for T1 (Figure 1D).

**Figure 2:**
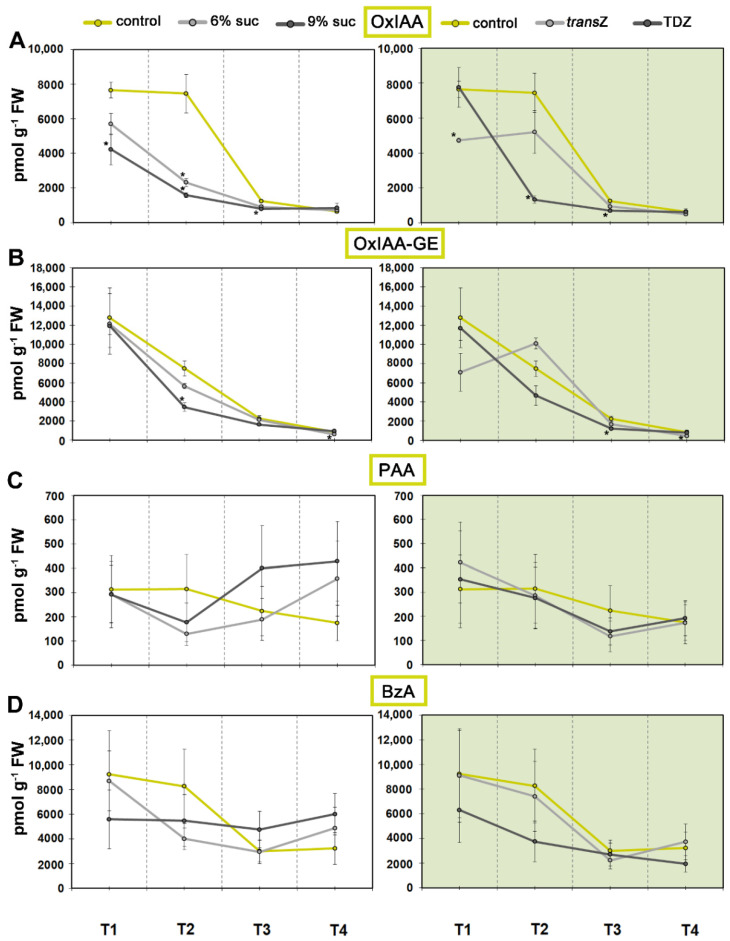
Changes in endogenous levels (in pmol g^−1^ FW) of indole-3-acetic acid (IAA) metabolites and phenolic auxin analogues at four developmental stages (T1–T4) during in vitro growth of kohlrabi (cv. Vienna Purple) on media supplemented with 3(control)/6/9% sucrose or 2 mg L^−1^ transZ/TDZ. (**A**) 2-oxindole-3-acetic acid (OxIAA); (**B**) oxo-IAA-glucose ester (OxIAA-GE); (**C**) phenylacetic acid (PAA); (**D**) benzoic acid (BzA). Results are expressed as mean ± SE (*n* = 3 independent biological replicates). Means marked with * are significantly different from control according to Student’s *t*-test (*p* < 0.05) at each developmental stage. *trans*Z: *trans*-zeatin; TDZ: thidiazuron.

On the other hand, high levels of 2-oxindole-3-acetic acid (OxIAA), an oxidation product of IAA and physiologically inactive IAA catabolite, were observed for T1 and T2, which was followed by a sharp decline (Figure 2A). Sugar treatment, particularly at 9%, resulted in a marked decrease in OxIAA, reaching levels close to control values at T4. Similar results were observed for TDZ, whereas *trans*Z had a statistically significant effect only at the beginning at T1. The most abundant IAA metabolite OxIAA-glucose ester (OxIAA-GE), the oxidation product of the glucose ester of IAA, also showed the highest value at the initial stage, followed by an almost linear decrease as development continued. TDZ and 9% sucrose further decreased the levels of this metabolite (Figure 2B). Levels of phenylacetic acid (PAA), a phenolic non-indole compound with weak auxin activity, remained relatively balanced and decreased slightly after T2 (Figure 2C). This decrease was more pronounced for the content of another phenolic compound, benzoic acid (BzA), but none of the treatments had a significant effect on either of the auxin analogues, PAA and BzA (Figure 2D).

**Figure 3:**
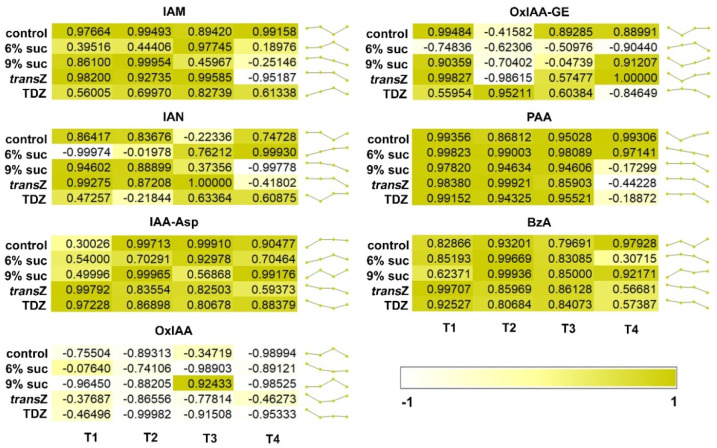
Pearson’s correlation coefficient (r) between the content of IAA and various auxin metabolites and/or analogues of kohlrabi (cv. Vienna Purple) seedlings treated with high sucrose concentration or cytokinins at four developmental stages (T1–T4).

Further analyses were performed to evaluate the correlation between the levels of different auxin metabolites and/or analogues and the content of IAA and its change during development (Figure 3). IAA-Asp and BzA all showed positive correlations with IAA regardless of the time point, but the levels were lower for CK-free media at T1 for IAA conjugate, and conversely for *trans*Z, TDZ and 6% sucrose treatments at T4 for BzA. The latter trend was more pronounced for PAA, where CK treatments and high sucrose resulted in negative correlation at the final stage. A similar situation was observed for IAM with negative values when 9% sucrose and *trans*Z were applied. The predominantly positive correlations were slightly shifted to negative for IAN, again with 9% sucrose and *trans*Z, but this time also with additional particular treatments during development. The relationship was even more altered with OxIAA-GE, while the generally strong negative correlation of OxIAA content with IAA levels was noted, interestingly with the exception of the 9% sucrose treatment at T3.

### 3.2. Cytokinins

**Figure 4:**
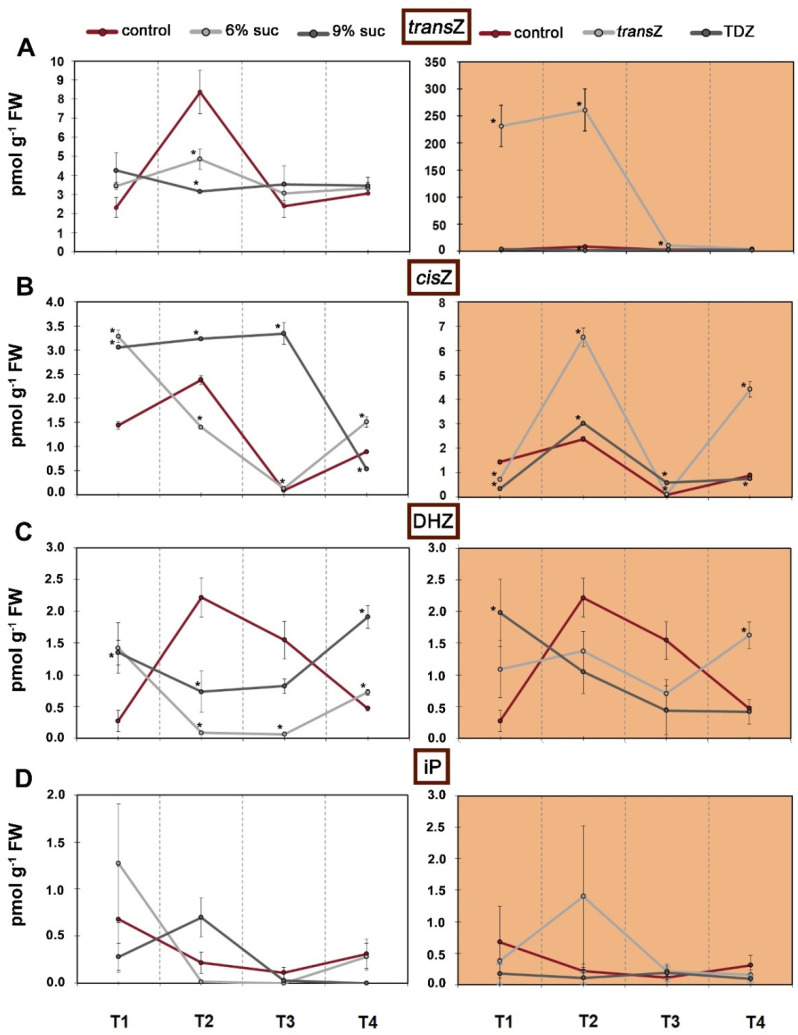
Changes in endogenous levels (in pmol g^−1^ FW) of free cytokinin bases at four developmental stages (T1–T4) during in vitro growth of kohlrabi (cv. Vienna Purple) on media supplemented with 3(control)/6/9% sucrose or 2 mg L^−1^ *trans*Z/TDZ. (**A**) *trans*-zeatin (*trans*Z); (**B**) *cis*-zeatin (*cis*Z); (**C**) dihydrozeatin (DHZ); (**D**) *N^6^*-(Δ^2^-isopentenyl)adenine (iP). Results are expressed as mean ± SE (*n* = 3 independent biological replicates). Means marked with * are significantly different from control according to Student’s *t*-test (*p* < 0.05) at each developmental stage. *trans*Z: *trans*-zeatin; TDZ: thidiazuron.

Focusing on bioactive forms of CKs, only CK free bases consisting of *trans*-zeatin (*trans*Z), *cis*-zeatin (*cis*Z), dihydrozeatin (DHZ) and *N^6^*-(Δ^2^-isopentenyl)adenine (iP) were analyzed in this study, as shown in Figure 4. More detailed profiles of particular CK types were provided in our previous work [39]. In untreated kohlrabi plants, the levels of *trans*Z and *cis*Z showed fairly similar patterns on a growth time scale, peaking at T2 (Figure 4A,B). This is also reported for DHZ (Figure 4C). The presence of higher sucrose concentrations (both 6% and 9%) in the growth media significantly lowered the content of *trans*Z and DHZ, while a later increase was observed for DHZ at the final growth stage. The opposite situation was observed for *cis*Z content when treated with 9% sucrose. It can be clearly seen that treatment with exogenous *trans*Z triggered an immense increase in endogenous concentrations of the same phytohormone at the earlier growth stages, T1 and T2, with subsequent shift toward control levels (Figure 4A), whereas TDZ had no significant effect regardless of the stage studied. Interestingly, a slightly different effect was observed for *cis*Z when maximum levels were reached at T2 and T4, and TDZ showed a similar but much less pronounced effect (Figure 4C). iP, which is the least abundant compared with other CK free bases, showed a decrease in content as the growth progressed, whereas none of the treatments applied caused statistically significant changes in its content (Figure 4D).

### 3.3. Relation between Auxins and Free Cytokinin Bases

**Figure 5:**
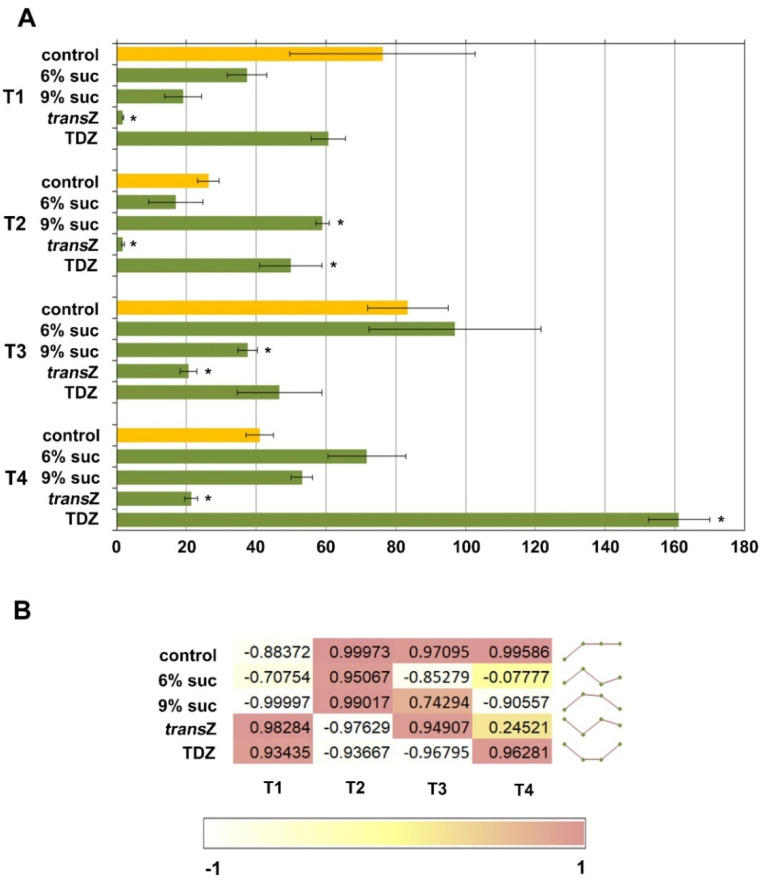
Relationship between endogenous auxin and CK free bases. Calculation of (**A**) ratio and (**B**) Pearson’s correlation coefficient (r) between the content of IAA and total CK free bases of kohlrabi (cv. Vienna Purple) seedlings treated with high sucrose concentration or cytokinins at four developmental stages (T1–T4). Results are expressed as mean ± SE (*n* = 3 independent biological replicates). Means of IAA/CK free base ratios marked with * are significantly different from control according to Student’s *t*-test (*p* < 0.05) at each developmental stage. *trans*Z: *trans*-zeatin; TDZ: thidiazuron.

The ratios between endogenous IAA and CK free bases were additionally evaluated and showed an alternating fluctuation as development progressed, with the value returning to the original level at T3 after an initial decrease at T2, and then decreasing again at the final stage (Figure 5A). As expected, the *trans*Z treatment significantly lowered the calculated ratio at all stages examined, consistent with the relatively high content of endogenous zeatin, particularly the *trans* isomer, in this treatment. With the highest contribution to the free bases, the *trans*Z content significantly affected the IAA/CK free bases ratio. Statistically significant changes compared with the control were also observed at T2 and T4 when TDZ was used, and at T2 and T3 after increasing sucrose concentration to 9%. However, these effects resulted in an increase in the ratio values above the control ones (Figure 5A).

A negative correlation between IAA and bioactive CK free bases was observed at the beginning in the control and sucrose treatments, shifting to a positive correlation after exogenous CKs were applied (Figure 5B). As development progressed, the situation completely reversed at T2, whereas a strong negative relationship was observed only in the treatments with 6% sugar and TDZ at T3 and 9% sucrose at T4.

### 3.4. Abscisic Acid

**Figure 6:**
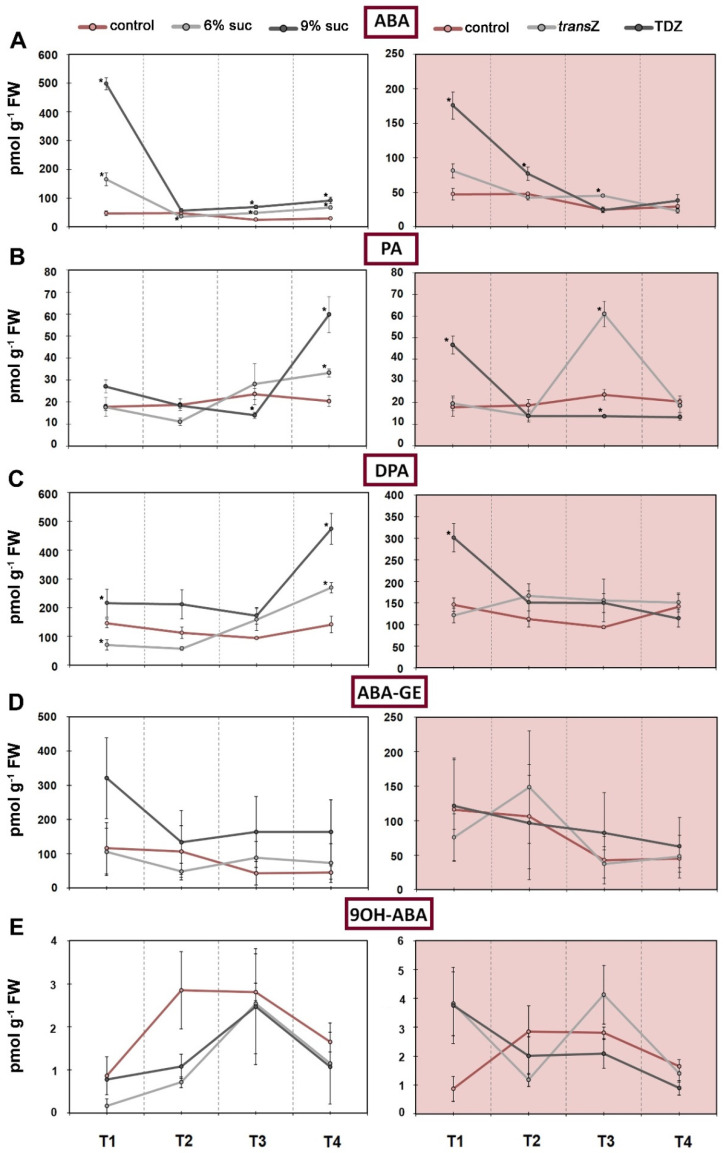
Changes in endogenous levels (in pmol g^−1^ FW) of abscisic acid (ABA) and related metabolites at four developmental stages (T1–T4) during in vitro growth of kohlrabi (cv. Vienna Purple) on media supplemented with 3(control)/6/9% sucrose or 2 mg L^−1^ *trans*Z/TDZ. (**A**) ABA; (**B**) phaseic acid (PA); (**C**) dihydrophaseic acid (DPA); (**D**) ABA-glucose ester (ABA-GE); (**E**) 9-hydroxy-ABA (9OH-ABA). Results are expressed as mean ± SE (*n* = 3 independent biological replicates). Means marked with * are significantly different from control according to Student’s *t*-test (*p* < 0.05) at each developmental stage. *trans*Z: *trans*-zeatin; TDZ: thidiazuron.

Abscisic acid (ABA) and its derivatives had more or less comparable profiles in control plants throughout development, with slight changes in content as growth progressed (Figure 6A–D). However, the levels of the ABA deactivation product 9-hydroxy-ABA (9OH-ABA) increased after the initial stage at T2 and T3, after which there was a decline (Figure 6E). High sucrose treatment, especially 9%, resulted in a sharp increase in ABA at T1, a sharp decrease at T2, reaching approximately control levels, and then moderately increased levels at other stages of the time scale (Figure 6A). A similar situation was found when CKs were applied, with TDZ showing the statistically significant increasing effect at T1. As for the ABA metabolite phaseic acid (PA), its accumulation was detected only at the last stage studied, T4, when increased sucrose was applied, whereas CKs differentially affected the levels of PA throughout development, implying that TDZ led to a significant increase at T1, followed by a further decrease below untreated levels, while *trans*Z caused a sharp increase at T3 (Figure 6B). Similar data as for PA were found for dihydrophaseic acid (DPA), ABA catabolite, except for the *trans*Z treatment, where no fluctuations were observed during early kohlrabi development (Figure 6C). In addition, no significant changes were found for 9OH-ABA and another ABA catabolite, ABA-glucose ester (ABA-GE), regardless of treatment type, as shown in Figure 6D and E, respectively.

**Figure 7:**
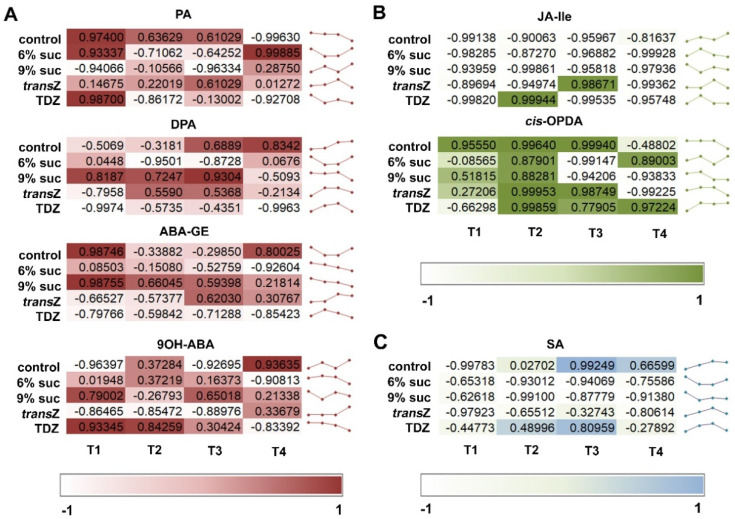
Pearson’s correlation coefficient (r) between content of (**A**) ABA and various ABA metabolites; (**B**) jasmonic acid (JA) and two JA metabolites; (**C**) JA and salicylic acid (SA) of kohlrabi (cv. Vienna Purple) seedlings treated with high sucrose concentration or cytokinins at four developmental stages (T1–T4).

Compared with the content of ABA, related metabolites showed a rather different correlation on a time scale that obviously depended on the treatment (Figure 7A). Each metabolite analyzed showed a specific pattern of changes. For example, although ABA and most of its derivatives generally showed comparable content profiles during development, PA and DPA, both deactivation products of ABA, had quite opposite correlation outlines, particularly at T1. This trend decreased as treatment continued.

### 3.5. Jasmonates and Salicylic Acid

**Figure 8:**
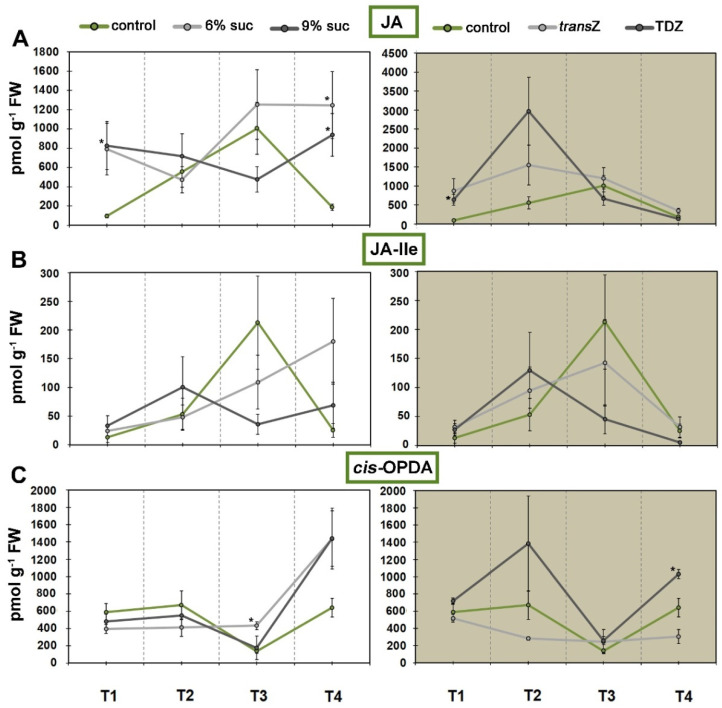
Changes in endogenous levels (in pmol g^−1^ FW) of jasmonic acid (JA) and related metabolites at four developmental stages (T1–T4) during in vitro growth of kohlrabi (cv. Vienna Purple) on media supplemented with 3(control)/6/9% sucrose or 2 mg L^−1^ *trans*Z/TDZ. (**A**) JA; (**B**) JA-isoleucine (JA-Ile); (**C**) *cis*-(+)-12-oxo-phytodienoic acid (*cis*-OPDA). Results are expressed as mean ± SE (*n* = 3 independent biological replicates). Means marked with * are significantly different from control according to Student’s *t*-test (*p* < 0.05) at each developmental stage. *trans*Z: *trans*-zeatin; TDZ: thidiazuron.

As for the stress-related hormones, jasmonic acid (JA) and its bioactive form (+)-7-iso-Jasmonoyl-L-isoleucine (JA-isoleucine; JA-Ile) showed a similar pattern of changes across growth stages, with concentrations increasing as development progressed, reaching a maximum at T3 and then decreasing back to initial levels at T4 (Figure 8A,B). Increasing the concentration of sucrose applied resulted in somewhat comparable changes. Whereas 6% sucrose generally caused an increase in values from T1 to T4, a concentration of 9% caused a decrease at T3. However, at the last stage examined (T4), there was a statistically significant increase in JA levels in both treatments compared with the control (Figure 8A). TDZ showed the same pattern in both phytohormones analyzed, with a significant difference at T1, whereas *trans*Z showed no statistical impact on JA and JA-Ile levels. In contrast, the content of the JA precursor, *cis*-(+)-12-oxo-phytodienoic acid (*cis*-OPDA), decreased in the non-treated plants at T3 compared with the other developmental stages, with statistically significant changes at T3 after treatment with a medium sucrose concentration (6%) and at T4 after TDZ application (Figure 8C).

Pearson’s correlation analysis revealed a remarkable negative correlation between JA and its bioactive form JA-Ile in almost all treatments during the four stages of kohlrabi in vitro development, except for the TDZ and *transZ* treatments in T2 and T3, respectively (Figure 7B). On the other hand, JA precursor *cis*-OPDA generally showed a positive correlation, especially in all treatments at T2, whereas the other stages showed both positive and negative values for the correlation coefficient depending on the treatment.

**Figure 9:**
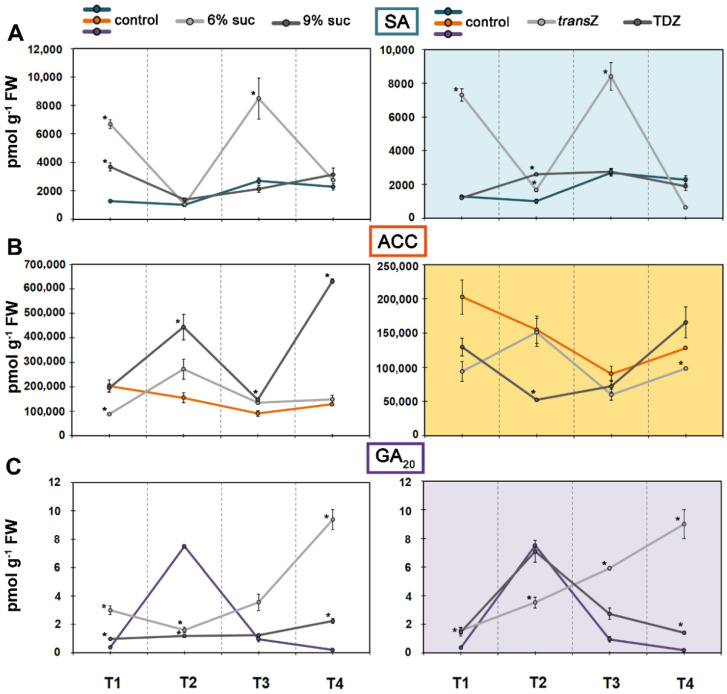
Changes in endogenous levels (in pmol g^−1^ FW) of (**A**) salicylic acid (SA); (**B**) 1-aminocyclopropane-1-carboxylic acid (ACC); and (**C**) gibberellin GA_20_ at four developmental stages (T1–T4) during in vitro growth of kohlrabi (cv. Vienna Purple) on media supplemented with 3(control)/6/9% sucrose or 2 mg L^−1^ *trans*Z/TDZ. Results are expressed as mean ± SE (*n* = 3 independent biological replicates). Means marked with * are significantly different from control according to Student’s *t*-test (*p* < 0.05) at each developmental stage. *trans*Z: *trans*-zeatin; TDZ: thidiazuron.

The T3 stage also showed the highest value for another stress-related hormone, salicylic acid (SA) (Figure 9A). Interestingly, the addition of 6% sucrose and *trans*Z resulted in almost the same SA profiles and led to a significant increase in the values of the first and third time points, reaching up to about 8500 pmol g^−1^ FW. Higher sugar concentrations and TDZ had a milder effect, with 9% sucrose leading to an increase in levels at T1 and the latter at T2.

JA and SA were predominantly negatively correlated, confirming the reciprocal interaction between these two important plant hormones, which usually influence each other through complex synergistic and antagonistic effects (Figure 7C).

### 3.6. 1-Aminocyclopropane-1-carboxylic Acid and Gibberellin GA_20_

The content of the direct ethylene precursor 1-aminocyclopropane-1-carboxylic acid (ACC) was the highest of all phytohormones studied and over time its content decreased, reaching the lowest value at T3. Both sugar concentrations elicited similar responses at the first three developmental stages, but an extraordinary increase was observed at T4 compared with the control (4.9-fold) as a result of the addition of 9% sucrose in the growth medium (Figure 9B). Exogenous CKs induced opposite changes that significantly decreased ACC content at T2 (by TDZ) and T4 (by *trans*Z).

Levels of gibberellin GA_20_ showed the highest value at T2 (Figure 9C). After treatment with elevated sucrose concentration, the measured values increased at the initial and final stages of growth but decreased at T2 compared with the untreated control. Treatment with *trans*Z had a similar effect to moderate sucrose concentration, while TDZ generally resulted in a similar profile to the control, with a single statistically significant increase at T4.

### 3.7. Principal Component Analysis

**Figure 10:**
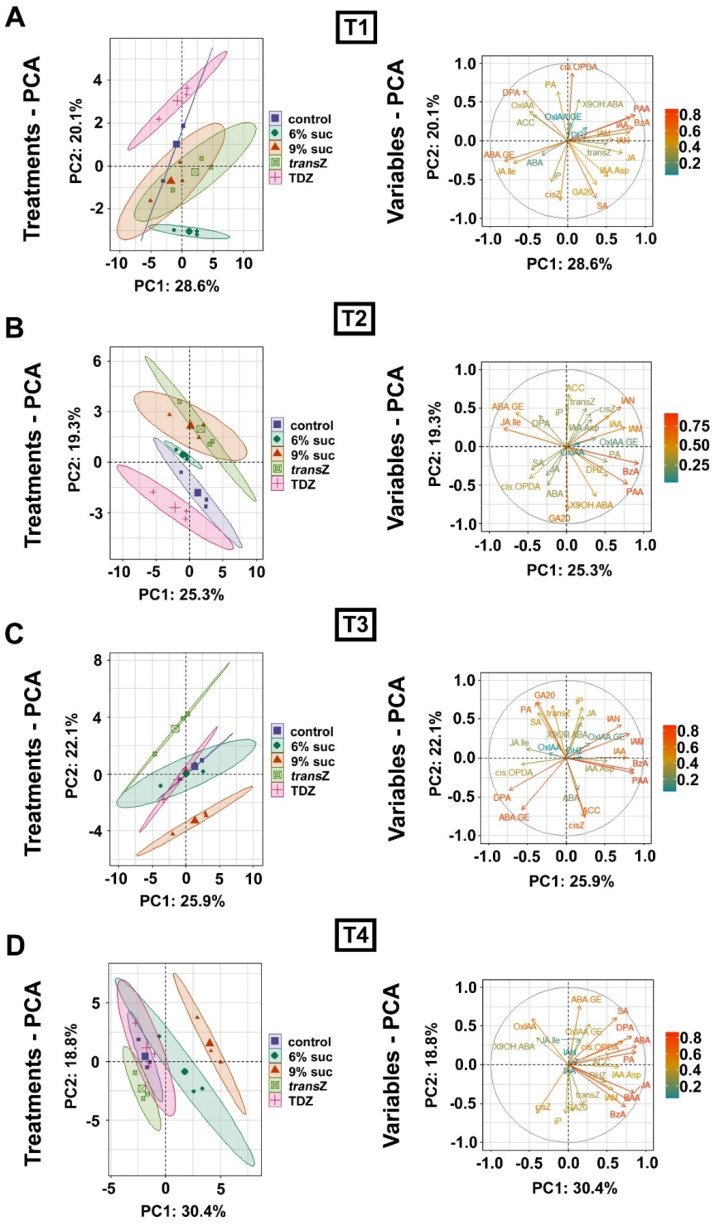
Principal component analysis (PCA) of the endogenous content of the phytohormone metabolites in kohlrabi (cv. Vienna Purple) during in vitro growth on media supplemented with 3(control)/6/9% sucrose or 2 mg L^−1^ *trans*Z/TDZ at four developmental stages: T1 (**A**); T2 (**B**); T3 (**C**); and T4 (**D**). The contributions of principal component 1 (PC1) and principal component 2 (PC2) to the overall variability of the measured data are indicated on the horizontal and vertical axes of the graphs, respectively. *trans*Z: *trans*-zeatin; TDZ: thidiazuron.

**Figure 11:**
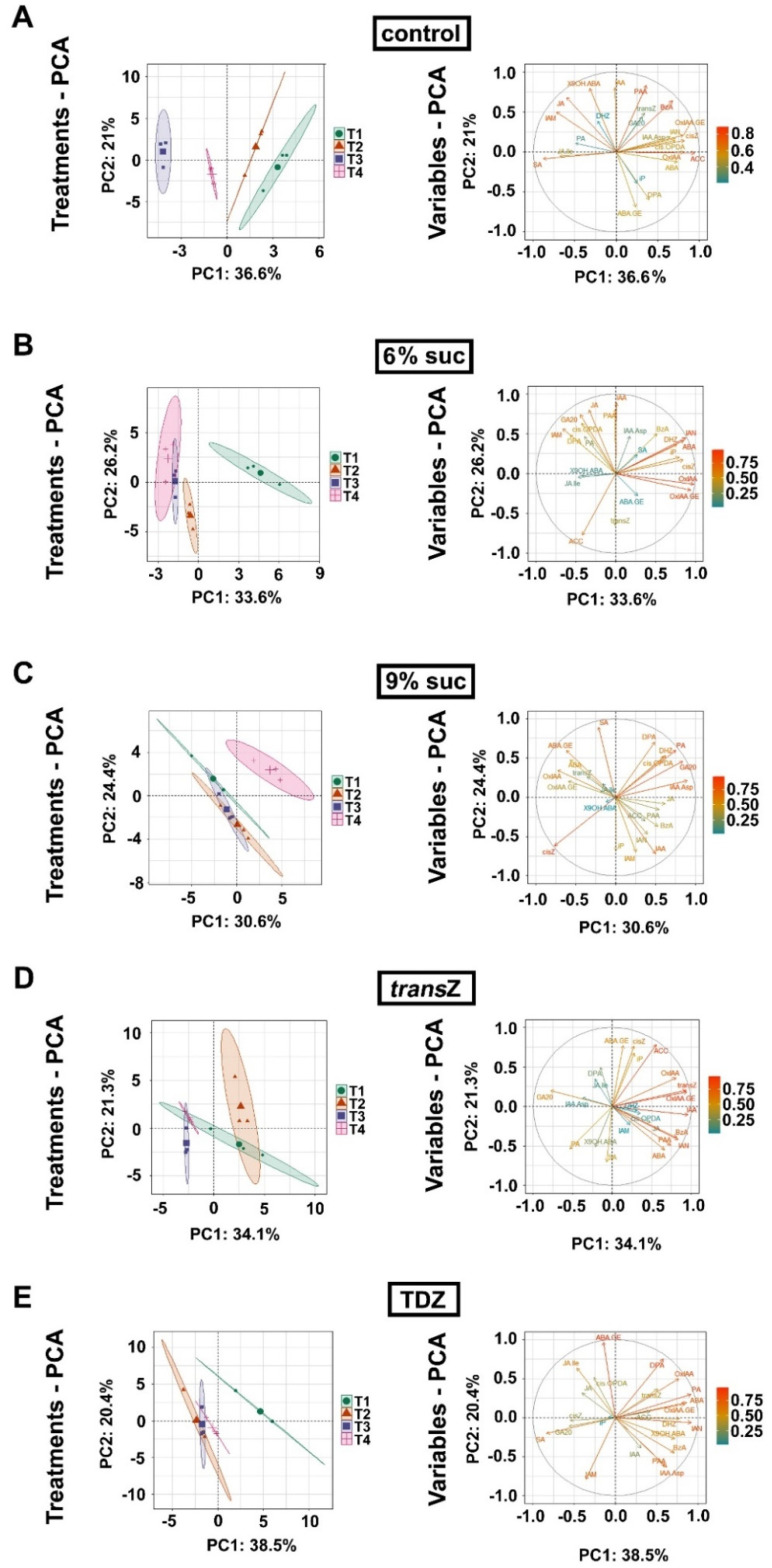
Principal component analysis (PCA) of the endogenous content of the phytohormone metabolites at four developmental stages (T1–T4) during in vitro growth of kohlrabi (cv. Vienna Purple) on media supplemented with 3% sucrose (control) (**A**); 6% sucrose (**B**); 9% sucrose (**C**); 2 mg L^−1^ *trans*Z (**D**); and 2 mg L^−1^ TDZ (**E**). The contributions of principal component 1 (PC1) and principal component 2 (PC2) to the overall variability of the measured data are indicated on the horizontal and vertical axes of the graphs, respectively. *trans*Z: *trans*-zeatin; TDZ: thidiazuron.

Principal component analysis (PCA) was performed to determine the major variability patterns of the kohlrabi phytohormonome between different culture treatments (Figure 10) and over the time-course of seedling development (Figure 11).

The distribution of data corresponding to the 6% sucrose treatments and the control shows a partial overlap over the entire course of seedling development (Figure 10), whereas the distribution of data corresponding to the 9% sucrose treatment differs significantly from the control starting at stage T2 (Figure 10B–D). The results for the *trans*Z treatment show an initial co-segregation with the data corresponding to the 9% sucrose treatment and an opposite pattern in the later stages. Interestingly, the distribution of data associated with the TDZ treatment largely matches that of the control data, especially at T3 and T4 stages, confirming that the TDZ treatment has the weakest effect on phytohormone content in kohlrabi seedlings, especially at the later stages (Figure 10C,D). The difference between the two CK treatments is remarkable at T1, T2, and T3 stages (Figure 10A–C), whereas this discrepancy is greatly reduced at the last stage of seedling development (Figure 10D). The variability of endogenous levels of particular phytohormone metabolites appears to be more or less random and does not seem to coincide with either of the two principal components of data variability during seedling development (eigenvector plots in Figure 10).

The PCA results for temporal variability of the kohlrabi phytohormonome are shown separately for each culture treatment in Figure 11. The distribution of the control data trends leftward along the principal axis of the major component, which accounts for 36.6% of the total data variability, from stage T1 to stage T3, whereas the hormonomic data at T4 show an inverse trend to the values measured at T2 (Figure 11A). While a major drift in the hormonomic data occurs between T1 and T2 in the 6% sucrose treatment, the variability in the phytohormonome from T2 to T4 appears to be minor (Figure 11B). Conversely, for the 9% sucrose treatment, the data for stages T1-T3 are clustered relatively close to each other, but a major drift occurs after T3, associated with a change in direction of data drift along the PCA matrix (T4 > T1 > T2 > T3) (Figure 11C). The distribution of data for the *trans*Z treatment within the PCA matrix appears to be more or less random, with the most pronounced data drift occurring from T2 to T3 (Figure 11D). Finally, a larger drift in the phytohormonome occurs at the T1–T2 transition in the TDZ treatment (Figure 11E). Regarding the variability of the endogenous content of certain phytohormone metabolites, the variability of ACC and OxIAA in the control treatment showed an important coincidence with the axis corresponding to principal component 1 (Figure 11A), whereas *trans*Z, IAA, and OxIAA-GE in the *trans*Z treatment showed variability close to the direction of PC1 (Figure 11D). In addition, the variability of IAA content was almost colinear with the PC2 axis in the control (Figure 11A) and in the 6% sucrose treatment (Figure 11B). The variability of *trans*Z was colinear in the opposite direction from IAA in the 6% sucrose treatment (Figure 11B), whereas endogenous iP-type CKs showed variability that was colinear with PC2 when 9% sucrose was applied (Figure 11C).

### 3.8. Sucrose and Cytokinin Interaction

**Figure 12:**
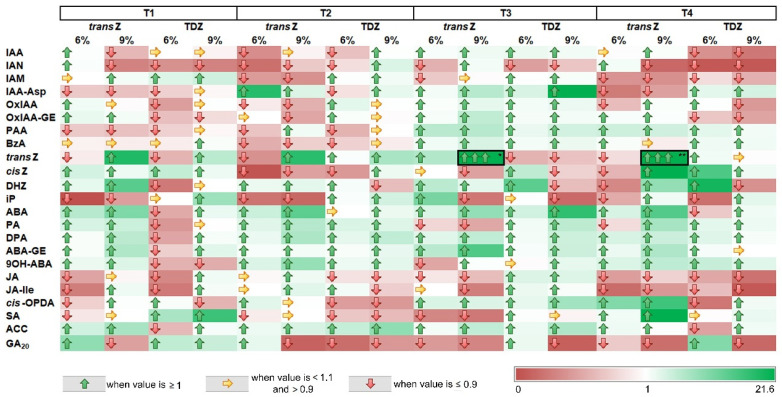
Heat map showing the relative abundance of the analyzed metabolites on treatments with both cytokinin (*transZ* or TDZ at 2 mg L^−1^) and high concentration of sucrose (6% or 9%) compared with the corresponding cytokinin-amended media with 3% sucrose. The ratios were calculated at four developmental stages (T1–T4) during in vitro growth of kohlrabi (cv. Vienna Purple). Additional arrows are provided for visual understanding of the results, and demonstrate arbitrarily set values (≥1; <1.1 and >0.9; ≤0.9); the color scale ranges from the minimum value (0) to the maximum value (21.6). */** The square boxes refer to two values (120.02 and 151.2, respectively) calculated above the set maximum; for interpretation reasons, these values are excluded when adjusting the color scale. *trans*Z: *trans*-zeatin; TDZ: thidiazuron.

In order to document the interplay of sucrose and CK treatments on kohlrabi growth and development in vitro, we cultured kohlrabi plantlets on media supplemented with both CK (*transZ* or TDZ at 2 mg L^−1^) and a high concentration of sucrose (6% or 9%) and determined the relative abundance of the analyzed metabolites in the treatments with elevated sucrose compared with the corresponding CK-amended media with 3% sucrose (Figure 12). The results show relationships depending on the metabolite, the growth stage, and the amount of sucrose applied. Phytohormones associated with IAA showed cyclic changes throughout development, with levels decreasing mainly when sucrose was elevated at the first two stages. The opposite was true at T3, where mainly IAA-Asp was increased and finally declined again at T4. As expected, the levels of ABA and related metabolites were greatly increased when sucrose was combined at high concentrations (especially 9%) with CKs in the growth media, regardless of developmental stage. The only significant exception to the observed trend was recorded at the T1 stage, when 6% sucrose was applied together with TDZ; all levels decreased. In general, this type of media resulted in a decrease in the content of almost all phytohormones analyzed at T1 (Figure 12). Another significant observation concerned the content of CK free bases, the levels of which were significantly elevated at the last two developmental stages studied (T3 and T4) when *trans*Z and 9% sucrose were added together to the growth medium. A massive increase of up to 150-fold was observed for endogenous *trans*Z levels, and a 20-fold increase was also revealed for *cis*Z. A noticeable increase was also noted for JA precursor *cis-*OPDA and SA when this treatment was used, also at T4 stage.

## 4. Discussion

In vitro studies of kohlrabi regeneration led to the discovery that indirect DNSO can be induced with exogenous CKs only, eliminating the need for auxin, while cultivation on PGR-free nutrient medium follows the normal developmental path of kohlrabi seedlings, without spontaneous callus and de novo shoot formation [36]. A correlation analysis between the level of endogenous phytohormones in the later DNSO phase and the regeneration potential of different kohlrabi explant types revealed that even in the absence of CIM, high endogenous auxin levels (and, more importantly, a high auxin/CK ratio) can be sufficient to induce callus formation, suggesting that the content of endogenous phytohormones is crucial for the induction of morphogenic events, rather than the composition of the culture media [36]. This finding raised the importance of another inadequately addressed issue in plant tissue culture: the relationship between exogenous application and endogenous phytohormone levels and the importance of phytohormone uptake from the media [45]. Equally important, subsequent studies found a significant effect of CK and sucrose treatments, including their interaction, in the course of kohlrabi DNSO, with a prominent increase in total endogenous CK levels after joint application of 2 mg L^−1^ *trans*Z and 9% sucrose, indicating possible involvement of sucrose in CK homeostasis and uptake [39].

For the first time, we provide a detailed analysis of the phytohormone profile of kohlrabi during seedling growth and development in vitro, encompassing induced DNSO. In addition, our study included the effect of CKs and high sucrose application (6% and 9%), revealing the hormonomics of kohlrabi.

The content of IAA and, to a greater extent, of the inactive IAA precursor IAM increased when kohlrabi plantlets grown on PGR-free medium reach the T3 developmental stage. This stage includes more intense development of true leaves and root system and an increase in stem height in control kohlrabi plantlets, so the observed elevation is consistent with the critical role of auxins in regulating root and leaf formation [46,47].

In addition to free IAA, conjugated IAA forms make up a larger proportion of plant auxins and represent primarily storage metabolites or degradative intermediates [3,48,49]. Due to conjugation, the plant can adjust the level of free auxin depending on current needs, thus contributing to auxin homeostasis. In contrast to IAA and IAM levels, the content of IAA-Asp decreased significantly in control kohlrabi plantlets at T2, indicating hydrolysis of conjugates and release of free IAA, which probably led to an increase in its content at T3. Moreover, the process of IAA oxidation to OxIAA plays a role in reducing IAA signaling in plants [50]. Therefore, a large decrease in OxIAA content at T3 argues for increased auxin activity at this stage.

Sugars modulate auxin biosynthesis, either by affecting the activity of auxin biosynthetic genes [51,52] or the concentrations of IAA precursors, thereby also promoting the accumulation of IAA conjugates [53]. This is consistent with our findings that higher levels of available sugars ultimately led to significant increases in IAA, IAM and IAN levels in developing kohlrabi. In addition, it has been suggested that auxin plays an important role in starch accumulation [54], leading to the possibility that excess amounts of sucrose taken up from growth media are processed and stored in kohlrabi plantlets.

The majority of auxin metabolites (IAM, IAN, PAA, BzA, IAA-Asp) mostly showed a positive correlation with the content of bioactive IAA, pointing out that these conversions and/or modifications occur proportionally to the bioactive IAA form and balance the metabolites and IAA. On the other hand, OxIAA is negatively correlated with IAA, indicating that oxidation of IAA removes IAA molecules from the active pool and irreversibly inactivates them [55]. However, under certain conditions, a discrepancy between these relationships could be observed; for example, IAM is negatively correlated with IAA at T4 only in *trans*Z treatment. This disturbed dynamic balance between IAA and IAM could be possibly the consequence of disturbance in the expression or activity of the enzymes responsible for conversion of Trp to IAA via IAM [56]. Additionally, a strong positive correlation of OxIAA and IAA was observed only at T2 when 9% sucrose was applied, suggesting that this particular stage and treatment allowed the presence of these two metabolites in constant ratio, apparently in a dynamic balance, during the noted stage.

CK free bases are generally considered to be bioactive CK forms [57] and usually account for only a small portion of total CKs in various plant species [58,59], including kohlrabi seedlings grown in vitro [36,39]. The most abundant CK free base in this study was *trans*Z, which is usually considered the most active of all [60,61,62]. In contrast to IAA, the content of zeatin-related nucleobases (*trans*Z, *cis*Z, DHZ), all of which have similar profiles on a growth time scale, decreased at the T3 stage of control kohlrabi after previously reaching its maximum at the T2 stage. Since the T2 stage of kohlrabi development is represented by plantlets bearing two young, rather small, developing true leaves, we can assume that bioactive CKs at this stage are fully involved in regulating various kohlrabi developmental processes, including the size and activity of the shoot apical meristem (SAM) [63].

A strong positive correlation of CK free bases with IAA content was observed at the last three developmental stages in control kohlrabi plantlets, after the value was initially negative, providing further evidence of their interaction, which is necessary for appropriate plant shaping. Previous literature data show that auxin and CKs affect each other’s endogenous levels and balance; this includes several mechanisms of interactions between these two groups of hormones that are essential during organogenesis [64,65,66,67,68,69,70]. Our results show that there is actually no universal rule during kohlrabi growth in vitro that could determine whether IAA and bioactive CKs are positively or negatively interrelated. There is often a significant correlation, but its direction depends on various factors, such as the developmental stage and the presence of sucrose and growth regulators in the growth media.

The ratio between endogenous IAA and CK free bases was highest at T4 under TDZ treatment, corroborating with our previous results [36]. This coincides with the development of the large callus at the bottom of the plantlet stem with developed de novo shoots [39]. The relationship between endogenous concentrations of IAA and CK free bases is considered an important indicator and prerequisite for the processes underlying callus induction and further shoot formation during the process of DNSO [71,72]. Although callus development occurs to a lesser extent in *trans*Z-treated kohlrabi [36], a similar change in the IAA/CK free bases ratio would be expected to be observed after *trans*Z application. However, this is not the case; the ratio is significantly lower compared to the control at all stages studied, which is the result of elevated endogenous levels of free bases, presumably due to the uptake of *trans*Z from the growth medium. Plant explants have previously been shown to be able to take up CKs from the in vitro growth medium, which consequently influence the endogenous content of CKs as well as other groups of phytohormones [73]. CK uptake by explants during DNSO is not yet fully elucidated, but it can be assumed to depend on several groups of CK transporters (reviewed in [45]).

Similar to the endogenous *trans*Z profile, the levels of GA_20_, the precursor of bioactive GAs [74], in control plants reached the maximum at T2, when true leaves emerged. Both *trans*Z and GA_20_ were detected in much lower amounts than IAA, but the recorded levels of these three important growth-related hormones in the control plants appeared to be balanced to allow proper development of kohlrabi. It is well known that the functions of GA and auxin coincide in controlling various processes during plant development, especially cell expansion and differentiation. Bioactive GAs affect plant growth by initiating the degradation of DELLA proteins, which belong to the family of nuclear growth repressors and act as key regulators in GA signaling [75,76]. Auxin levels control the amount of DELLA proteins, which in turn are involved in integrating signals from ethylene, auxin, and GAs during growth [77,78]. Recent findings confirmed that, in general, low levels of GAs are a prerequisite for maintaining the undifferentiated status of vegetative SAM, but high levels of gibberellin are required for later SAM development [79].

The TDZ treatment showed a similar profile to the control, with a marked decrease in GA_20_ content at T3 and T4 after the maximum at T2. This decrease is necessary for shoot regeneration, on which gibberellins have an inhibitory effect through negative regulation by morphogenic gene *SHOOTMERISTEMLESS* (*STM*) [80,81]. The striking pattern is disturbed under both elevated sucrose and *trans*Z treatment, with GA_20_ levels increasing continuously, even during shoot regeneration, which may be due to a disturbance in phytohormone homeostasis. In addition, sucrose treatment was shown to be negatively correlated with gibberellins [82]; however, GA_20_ levels in kohlrabi were significantly higher at T4 than in the control despite their previous decrease in content.

Like other stress-related hormones, such as JA, SA, and ethylene, ABA has been shown to serve a dual function by regulating various processes of plant growth and development [83]. While ABA and most related metabolites varied only slightly during development of control kohlrabi, sucrose application resulted in an increase in the levels of ABA, PA and DPA at T4 as well as of ethylene precursor ACC. Significant interactions were found between the sugar and ABA/ethylene signaling pathways [84]. In contrast to the negative feedback loop in gibberellin biosynthesis [85], a positive feedback loop has been proposed in the regulation of ABA biosynthesis [86,87], indicating specific sugar adjustment of ABA biosynthetic genes and ABA accumulation.

The content of JA and its biologically active form JA-Ile was significantly increased at T3. However, the content of the JA precursor, *cis*-OPDA, reached a minimum at the same stage, supporting the increase in the levels of active jasmonates. Plants have different strategies to regulate the production and activity of JA, including controlling the activity of enzymes in the JA biosynthetic pathway and accumulating or reusing jasmonate intermediates or conjugates when JA is overproduced or a rapid JA response is required [88].

A strong negative correlation between JA-Ile and JA was observed throughout development, regardless of stage and treatment, indicating the lack of dynamic balance between these two metabolites and, rather, the process of JA conjugation “draining” certain content of JA molecules from its assembly. As with IAA and related metabolites, the exceptions on *trans*Z at T3 and on TDZ at T2 were present with strong positive correlation, with coefficient values very close to 1, implying the reversal of the dynamic relationship between JA and JA-Ile by various aspects of growth, including the availability of sugar, PGRs, as well as the developmental stage.

In kohlrabi control plantlets, SA content was also increased at the T3 stage, as confirmed by the positive correlation with JA, although this increase was smaller compared with JA. As kohlrabi plantlets progress to the T3 stage, they grow intensively, develop a root system and true leaves, consume necessary nutrients from the growth medium, excrete the products of their metabolism, and release ethylene into the surrounding atmosphere, which might be related to a decrease in the content of ACC, a precursor of gaseous ethylene. All of this could presumably cause a stressful environment for the plantlets, triggering the observed increase in JA and SA contents at T3. However, this assumption does not explain the lack of the expected ABA response. Perhaps the answer lies in a more rapid response of JA and SA.

Supplementation of the growth media with sucrose or CKs elicited significant changes in the concentrations of most phytohormone groups at each kohlrabi developmental stage compared with the control. To better interpret the results, we performed PCA, which showed that sucrose treatment, especially 9%, had a stronger effect on endogenous hormone levels than treatments with CK.

As mentioned above, CKs alone are sufficient to induce DNSO in kohlrabi seedlings. Their uptake from the growth media affects not only the endogenous content of CKs but also other phytohormone groups studied. PCA analysis showed that the distribution of data related to TDZ treatment was highly consistent with the distribution of control data, particularly at T3 and T4 stages. Thus, the TDZ treatment had the weakest effect on phytohormone content of kohlrabi seedlings, which was especially pronounced at the later stages. Moreover, stages T1 to T3 were characterized by significant dissimilarity between the two CK treatments. However, in the last phase, the observed difference decreased markedly. Our previous studies showed that treatments with synthetic CK, such as TDZ, generally contributed less to the endogenous CK levels of kohlrabi plantlets and also decreased the content at certain stages compared with treatment with the naturally occurring cytokinin, *trans*Z [36,39]. Not surprisingly, treatment with *trans*Z caused the most striking change in the form of an increase in the endogenous levels of zeatin-type nucleobases, especially those of the same type with which the growth medium was supplemented. Thus, exogenous application affected CK homeostasis, resulting in altered interactions of endogenous CKs.

We can assume that the observed dissimilarity of the two CK treatments is due to differences in their mode of action. The mechanism of action of TDZ is not yet fully understood; however, it is known to have analogous effects to CKs and the ability to competently bind CK receptors [89]. In addition, TDZ inhibits CK oxidase/dehydrogenase (CKX) [90,91,92] and may interact with signaling pathways of other phytohormones, triggering their down- or up-regulation [93]. Furthermore, it was shown during the induction and formation of calli in *Scutellaria baicalensis* Georgi, that higher TDZ concentrations (1–5 mg L^−1^) could decrease the endogenous levels of benzyladenine (BA) and IAA [94].

It has already been shown that increased sucrose content in the growth media affects the total CK levels in kohlrabi [39], and significant changes in other phytohormones were also observed in the present study. PCA revealed that in the 6% sucrose treatment, the main differences in the kohlrabi phytohormonome occurred between stages T1 and T2, but further variability from T2 to T4 appeared to be insignificant. However, when treated with 9% sucrose, the hormonomic data for the first three stages were quite close, but greater variability occurred at the last stage.

The use of sugars is mandatory in in vitro culture because growing plants utilize sugars primarily as a carbon source, although they also act as osmotic regulators to some extent. Their role as an energy source has been shown to be independent of their effects on plant developmental processes [95]. Moreover, sugar concentrations other than optimal have been shown to induce various alterations in physiological and morphological processes during plant growth and development [33,96,97]. Our previous experiments have shown that the optimal sucrose concentration for in vitro cultivation of kohlrabi is 3% [35,36,38], so it is not surprising that increasing the amount of available sugar in the present study affected phytohormone homeostasis of kohlrabi. However, it appears that different results are observed depending on the amount of sucrose supplied, indicating that plants have a complex machinery responsible for fine-tuning their responses to changing growth conditions. It is possible that young seedlings are more sensitive to a moderate (6%) sucrose concentration by altering their hormonal homeostasis, while older plants may be less sensitive. In contrast, a sucrose level of 9% in the growth medium is quite high and very stressful and potentially harmful for growing plants that need longer to adjust their metabolism.

Joint application of CKs and high sucrose resulted in various changes in the abundance of different phytohormones depending on the type of metabolite, growth stage, and sucrose concentration applied. Most striking was the increase in the content of zeatin nucleobases, especially *trans*Z at T3 and T4, when exogenous *trans*Z and 9% sucrose were applied together, confirming our previous results [39].

Sugars and CKs are considered key regulators of many aspects of plant life, but their interplay is very complex and is adjusted for optimal plant response to exogenous and endogenous signals. They can act both synergistically and antagonistically depending on the nature of the physiological process, but also affect each other in terms of signaling and content [98]. It was reported that glucose induces accumulation of CKs by activating *IPT3* expression and CKX4 repression in *Arabidopsis* [99]. Hence, the accessibility of high amounts of sucrose in growth media could potentially alter the uptake of exogenous *trans*Z, or the assimilation of excessive sucrose itself could affect the specific signaling pathways, ultimately leading to an increase in endogenous zeatin content. Plants are indeed capable of recognizing various soluble sugars, and they have several sugar-related signaling pathways, including hexokinase (HXK), regulator of G-protein signaling (RGS1), sucrose-nonfermentation1-related protein kinase1 (SnRK1), and target of rapamycin (TOR) kinase [98,100]. Which of these elements interfere with *trans*Z pathways to cause such strong accumulation remains to be fully elucidated.

## 5. Conclusions

In this study, we established for the first time the complete phytohormone profiles of in vitro grown kohlrabi (*Brassica oleracea* L. *var. gongylodes*) seedlings over four stages of early growth and shoot regeneration, and revealed the effects of CK (*trans*-zeatin or thidiazuron, applied at 2 mg L^−1^) and high sucrose concentration (6% or 9%) on the hormone profiles of kohlrabi plantlets. We showed that a number of phytohormones and their metabolites exhibit complex patterns of change in endogenous levels over time, and that exogenously supplied CKs or high sucrose concentrations also affect these patterns in a very complex manner, depending strongly on the developmental stage of the plantlets. In general, high sucrose concentration caused more pronounced changes in phytohormonal profiles of kohlrabi than exogenously supplied CKs, while among CKs, *trans*Z generally caused more pronounced changes in phytohormonome than TDZ. In addition, analysis of the correlation between the levels of certain bioactive forms of CKs and their precursors or metabolites revealed that the dynamic balance between a bioactive hormone and its metabolite at a particular developmental stage can be disturbed or restored by treatment with CK or sucrose. Thus, the correlation between the levels of two molecular forms of a phytohormone can change from strongly positive to strongly negative and vice versa. The crosstalk of hormone signaling pathways appears to be important in modulating the response to sugars and the function of specific hormones and/or their metabolites at different sugar concentrations in kohlrabi seedling development and shoot regeneration.

## Data Availability

Not applicable.

## Supplementary Materials

The following supporting information can be downloaded at: https://www.mdpi.com/article/10.3390/life12101585/s1, Figure S1: Molecular structure of analyzed phytohormone groups: (A) auxins; (B) cytokinin nucleobases; (C) abscisic acid; (D) jasmonates; (E) salicylic acid; (F) gibberellin; and (G) ethylene precursor.
