# Supplementary material for: Comprehensive Phytohormone Profiling of Kohlrabi during In Vitro Growth and Regeneration: The Interplay with Cytokinin and Sucrose
Source: Life (Basel). 2022 Oct 12;12(10):1585. doi: 10.3390/life12101585 (PMC9604816; doi:10.3390/life12101585)
Supplement: Supplementary file 1 [file life-12-01585-s001.zip › life-1941133-supplementary.pdf]

life

MDPI

Supplementary Materials for Comprehensive Phytohormone Profiling of Kohlrabi during In Vitro Growth and Regeneration: The Interplay with Cytokinin and Sucrose

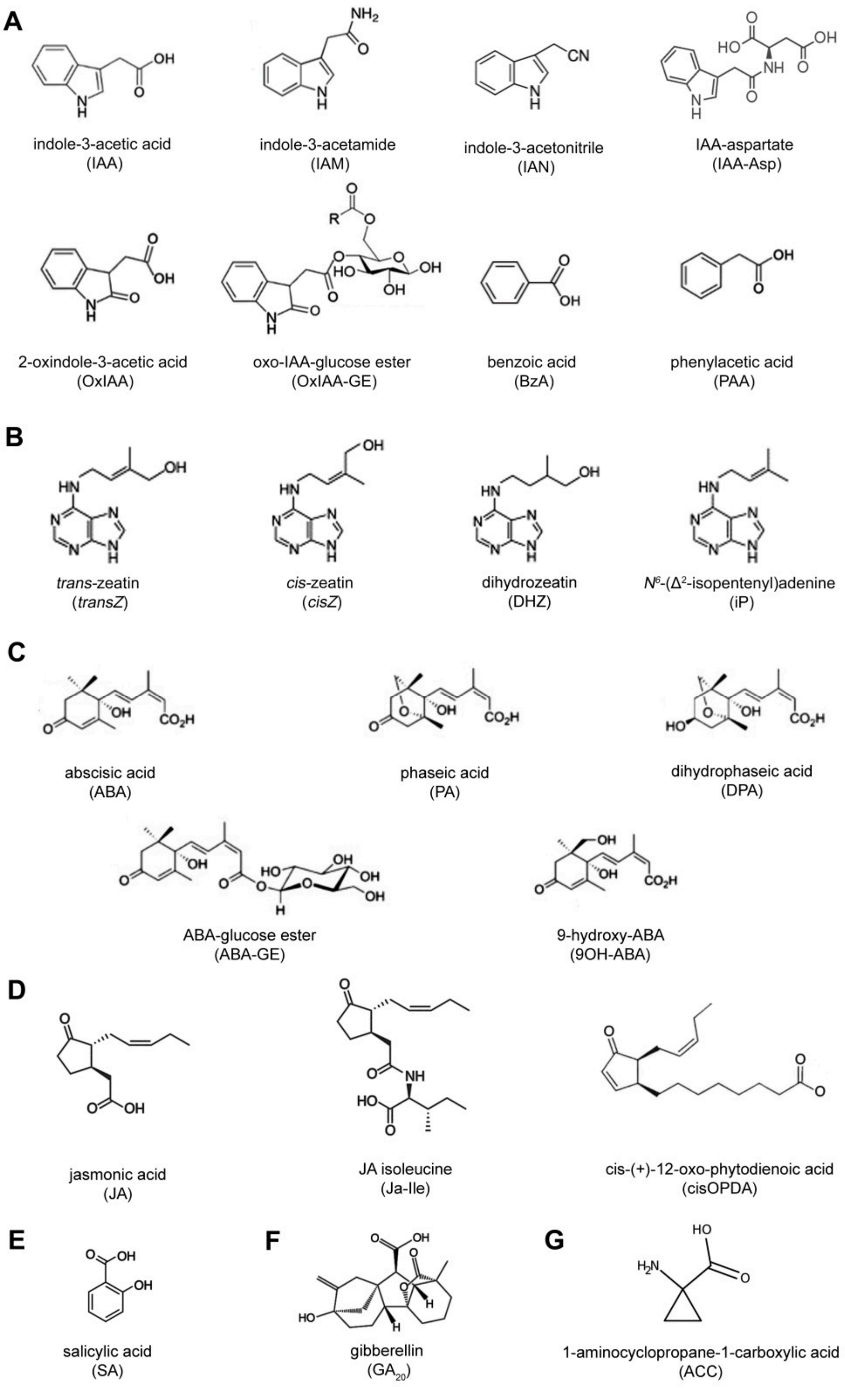

Figure S1. Molecular structure of analysed phytohormone groups: (**A**) auxins; (**B**) cytokinin nucleobases; (**C**) abscisic acid; (**D**) jasmonates; (**E**) salicylic acid; (**F**) gibberellin; and (**G**) ethylene precursor.
